# COVID-19 Syndemic: Convergence of COVID-19, Pulmonary Aspergillosis (CAPA), Pulmonary Tuberculosis, Type 2 Diabetes Mellitus, and Arterial Hypertension

**DOI:** 10.3390/diagnostics12092058

**Published:** 2022-08-25

**Authors:** Jose Isaias Badillo-Almaraz, Sergio Andres Cardenas-Cadena, Fausto Daniel Gutierrez-Avella, Pedro Javier Villegas-Medina, Idalia Garza-Veloz, Valentin Badillo Almaraz, Margarita L Martinez-Fierro

**Affiliations:** 1 Molecular Medicine Laboratory, Unidad Academica de Medicina Humana y C.S., Campus UAZ siglo XXI-L1, Universidad Autonoma de Zacatecas, Zacatecas 98160, Mexico; 2 Hospital General Luz Gonzalez Cosio, Circuito ciudad Gobierno, Zacatecas 98160, Mexico; 3 Clinica San Antonio Memorial Center, Rio Grande, Zacatecas 98400, Mexico; 4 Unidad Academica de Estudios Nucleares. Universidad Autonoma de Zacatecas, Zacatecas 98000, Mexico

**Keywords:** syndemic, *Mycobacterium tuberculosis*, invasive pulmonary aspergillosis, COVID-19-associated pulmonary aspergillosis, opportunistic infections, non-communicable diseases

## Abstract

Bacterial coinfections, which increase the severity of respiratory viral infections, are frequent causes of mortality in influenza pandemics but have not been well characterized in patients with Coronavirus disease 2019 (COVID-19). Moreover, the association of COVID-19 infection with pulmonary *Mycobacterium tuberculosis* disease (TB) and concurrent pulmonary fungal infection is not well known. The classification of patients with COVID-19-associated pulmonary aspergillosis (CAPA) using the current definitions for invasive fungal diseases has proven difficult. In this study, we aimed to provide information about three patients with underlying diseases ongoing with COVID-19 and co-infection with pulmonary TB, and with COVID-19-associated pulmonary aspergillosis (CAPA). At the time of hospital admission, each patient presented complications such as decompensated T2DM with diabetic ketoacidosis and/or hypertension. Findings of chest computed tomography and serum galactomannan by radioimmunoassay were useful for classifying them as possible CAPA. One of the three possible CAPA cases was fatal. These three cases are rare and are the first of their kind reported worldwide. The generation of reliable algorithms, early diagnosis, standardization of classification criteria, and the selection of specific and personalized treatments for COVID-19-associated opportunistic infections, including CAPA, are necessary to improve outcomes in these kinds of patients.

## 1. Introduction

A syndemic is a set of linked health problems involving two or more afflictions that interact synergistically and contribute to an excessive burden of disease in a population by person, place, or time [1]. The term “syndemic” describes how epidemics can overlap with one another and the social and cultural problems that ensue [1]. The recent coronavirus disease 2019 (COVID-19) crisis is a syndemic because two categories of disease are present, involving infection with severe acute respiratory syndrome coronavirus 2 (SARS-CoV-2) and an array of non-communicable diseases (NCDs). In Mexico, the number of patients with NCDs (i.e., diabetes mellitus (DM), obesity, systemic arterial hypertension (SAH), etc.) is increasing, as is their susceptibility to pathogens [2]. Bacterial co-infections increase the severity of respiratory viral infections and are frequent causes of mortality in influenza pandemics; however, they have not been well characterized in patients with COVID-19, and neither has their association with pulmonary tuberculosis disease nor pulmonary fungal infection such as concurrent Aspergillosis.

### 1.1. Pulmonary Microbial Infections

The presence of bacterial co-infections has been reported in about 4% of patients with respiratory viral infections upon hospital admission. *Staphylococcus aureus* (*S. aureus*), *Streptococcus pneumoniae* (*S. pneumoniae*), and *Haemophilus influenzae* (*H. influenzae*) are the most common pathogens, while atypical bacteria are rare, as is the association with pulmonary tuberculosis (TB).

#### 1.1.1. Invasive Pulmonary Aspergillosis (IPA)

IPA is a life-threatening fungal infection, typically occurring in severely immunocompromised patients with prolonged, profound neutropenia, *Aspergillus fumigatus* being the most frequent etiological agent. The delay in IPA diagnosis is one of the main causes of its high lethality. Cases of influenza-associated pulmonary aspergillosis (IAPA) were reported in up to 19% of patients with influenza in the intensive care unit (ICU). The mortality rate of IAPA was 51%, compared to 28% in influenza patients without IAPA [3].

#### 1.1.2. COVID-19-Associated Pulmonary Aspergillosis (CAPA)

CAPA, the COVID-19/IPA infection, is a complex disease involving a continuum of *Aspergillus* respiratory tract colonization, tissue invasion, and angioinvasion in a COVID-19 positive patient [4]. This condition has been reported to have a similar mortality rate than IAPA (52–51%, respectively); however, there are some differences between the two, including typology and comorbidities, viral effects, tissue tropism, the host immune response to the pathogen, the results of diagnostic tests for *Aspergillus* and the Galactomannan (GM), a conidia cell wall component, that can be used as a biomarker to stage CAPA infection. In December 2020, a consensus case definition of CAPA was issued by the European Confederation for Medical Mycology (ECMM) and the International Society for Human and Animal Mycology (ISHAM), categorizing CAPA patients as proven, probable, and possible [4,5]. However, this category differs fundamentally from that proposed by the European Organization for Research and Treatment of Cancer (EORTC)/Mycosis Study Group Education and Re-search Consortium (MSGERC), which leads to the important issue of the lack of a universal consensus for CAPA operational case definition.

#### 1.1.3. Pulmonary Tuberculosis (PTB)

PTB is an ancient human infectious disease caused by *Mycobacterium tuberculosis* (Mtb) and principally affects the lungs. It is a major cause of death worldwide and was declared a global health emergency by the World Health Organization (WHO) in 1994. The annual number of cases is increasing: approximately one-third of the world’s population is thought to be infected with the causative organism, Mtb. According to estimations, a quarter of the world’s human population has latent TB infection [6]. Among individuals with latent Mtb infection and no underlying medical problems, reactivation disease occurs in 5–10% of cases. The risk of TB reactivation is markedly increased in patients with human immunodeficiency virus (HIV) infection [7,8].

**Figure 1:**
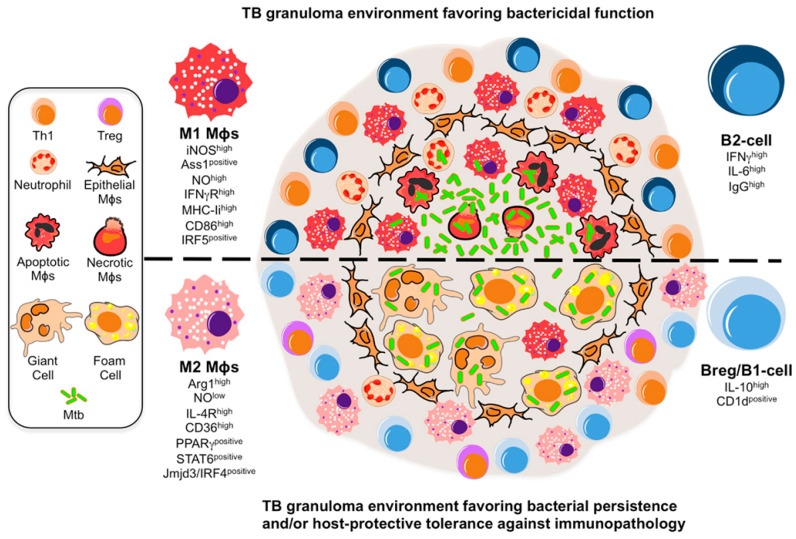
Model illustrating the tuberculosis (TB) granuloma environment and the roles of B cells T cells, and macrophages during TB granuloma formation. Taken from Lugo-Villarino et al. (2012) [14], distributed under the terms of the Creative Commons Attribution License https://creativecommons.org/licenses/by/3.0/; accessed date: 20 May 2022.

Mtb has no known environmental reservoir and the principal mode of Mtb spread is through inhalation of infected aerosolized droplets [9,10,11]. Inhalation of Mtb leads to one of four possible outcomes: 1. Immediate clearance of the organism; 2. Latent infection; 3. The onset of active disease (primary disease); and 4. Active disease many years later (reactivation disease). Infectious droplet nuclei are deposited in the alveolar spaces of the exposed person. The infectious dose for a person is reported to be between 1 and 200 bacilli; however, as a single aerosol droplet can contain anywhere from 1 to 400 bacilli, it is unclear what is considered a biologically relevant dose [12]. Mtb can be phagocytosed by alveolar macrophages, epithelial cells, dendritic cells (DC), and neutrophils. Though tubercle bacilli are killed by alveolar macrophages, they can also kill macrophages through apoptosis. These macrophages are stimulated by ligation of TLR-2, TLR-4, and other PRRs to produce proinflammatory cytokines and chemokines, such as TNF-α, IL-1b, IL-6, and IL-12. TNF-α is a critical cytokine in organized granuloma formation (Figure 1), driving the recruitment of more leukocytes to the site of infection [10,13,14].

Unlike other bacterial pathogens, Mtb does not have classical virulence factors such as the toxins produced by *Corynebacterium diphtheriae, Escherichia coli O157:H7, Shigella dysenteriae*, and *Vibrio cholerae*. While there is limited knowledge of how Mtb causes disease, its virulence can be measured and an important parameter that is usually associated with Mtb virulence is the bacterial load or burden, i.e., the number of bacteria found in the infected host after the initial infection. In addition, Mtb virulence mutants that have lower bacterial loads during animal infections exhibit different growth curves during this process [10,15]. The gold standard for TB diagnosis is culture of Mtb, and molecular diagnostic tests such as Gen Xpert and Gen Xpert Ultra take principal place in the diagnosis of treatment-sensitive and treatment-resistant MTb.

### 1.2. COVID-19 and Comorbidities

Comorbidities are defined as one or more conditions that exist simultaneously, independently or not, with a disease considered as primary [16]. Diabetes (hyperglycemia) increases the risk for infections as reported during previous outbreaks. Since COVID-19, people with diabetes have shown poorer prognoses, likely due to the multifactorial and syndromic nature of diabetes [17]. An unusually high number of patients with COVID-19 developing diabetic ketoacidosis (DKA) or hyperglycemic hyperosmolar syndrome (HHS) has been noted. Poor outcomes of COVID-19 were reported for two clinical cases of DKA and HHS; a direct effect of SARS-CoV-2 was postulated, since the virus binds to the ACE2 receptor, which (among other tissues) is expressed in β-cells of pancreatic tissue. β-cell infection by SARS-CoV-2 may result in an acute loss of insulin secretory capacity, along with a stress condition, and the CSS could lead to a metabolic deterioration with subsequent development of DKA or HHS. This is likely to increase the risk of thrombosis, which is related to severe COVID-19 [17].

Both diabetes and obesity are characterized by chronic, low-grade inflammation with increased concentrations of pro-inflammatory leptin, reduced anti-inflammatory adiponectin, and gut dysbiosis, which might increase the inflammatory response to infection by SARS-CoV-2 [17,18,19]. Several mechanisms are associated with obesity and worse COVID-19 illness and/or outcomes [19]. The first and best known is related to the detrimental restrictive ventilatory effect of abdominal fat. Second, respiratory dysfunction in patients with severe COVID-19 might depend on impaired lung perfusion due to intravascular disseminated coagulation, because obesity and diabetes are prothrombotic conditions, and pulmonary embolism was the direct cause of death. Immune dysregulation and chronic inflammation are associated with obesity and may also mediate progression toward COVID-19-associated organ failure [19]. In the same way, myocarditis and cardiomyocyte dysfunction could be worsened by local biological effects of epicardial adipose tissue because the volume of epicardial adipose tissue is directly associated with BMI. Furthermore, ACE2 is highly expressed in the epicardial adipose tissue of patients with obesity, and may promote adipocyte viral infection and enhance TNF-α and IL-6 release.

Systemic arterial hypertension (SAH) is the most frequent comorbidity observed in patients with COVID-19 [6,16] and is associated with poorer prognosis [17]. Since SARS-CoV-2 binds to ACE2 to enter target cells, the high prevalence of severe infection in patients with arterial hypertension may be related to the use of ACE inhibitors. Previous reports proposed that ACE inhibitors and angiotensin receptor blockers would increase the expression of ACE2, and that their use may promote target cell infection and disease progression [20]. However, given its structural differences with ACE, ACE2 does not represent a target of these drugs. Furthermore, the interaction of ACE inhibitors with the renin–angiotensin system is not completely understood [17]. An increase in angiotensin II expression was induced by infection with SARS-CoV-2, and a subsequent downregulation of ACE2 was observed. These events induce a loss of anti-inflammatory effects in the respiratory tract, resulting in alveolar wall thickening, edema, inflammatory infiltration, and bleeding. A favorable effect of ACE inhibitors and angiotensin receptor blockers on the risk of community-acquired pneumonia has been suggested [18,19,21]. In cases of acute myocarditis associated with COVID-19, direct myocardial injury has been postulated as the cause of myocarditis [22], but evidence of myocardial injury may be indirect.

### 1.3. Aim of the Study

The aim of the study was to provide information about three patients with ongoing COVID-19, co-infection with pulmonary Mtb disease, and possible CAPA, each one with comorbidities of NCDs such as decompensated type 2 diabetes mellitus (T2DM) with moderate DKA and HHS, in accordance with criteria of the American Diabetes Association (ADA [23], and SAH grade I, in accordance with the criteria of the International Society of Hypertension (ISH) [24], diagnosed and managed in hospital for the first time.

## 2. Materials and Methods

### 2.1. Patients

This study was approved by the Ethics and Research Committee CONBIOETICA-32-CEI-001-20180807 of the General Hospital Zacatecas Luz Gonzalez Cosío, with registration number 0266/2021/C. The patients, who had COVID-19, probable CAPA, pulmonary TB, and NCDs such as decompensated T2DM with moderate DKA, were recruited from May to Aug 2021 in the General Hospital of Zacatecas, a public health center. Only one patient was recruited in a private health center.

### 2.2. Definition of CAPA (2020 ISHAM/ECMM Consensus Definitions)

For this study, CAPA was defined as IPA in temporal proximity to a preceding SARS-CoV-2 infection, positive SARS-CoV-2 real-time polymerase chain reaction (RT-PCR) anytime during the 2 weeks between hospital admission and ICU admission, or positive RT-PCR within 72–96 h of ICU admission. CAPA might then develop, usually during the following weeks. To define and classify CAPA, the 2020 ISHAM/ECMM consensus criteria were mainly considered as the basis; however, we included some other considerations as outlined in the following paragraphs. As published previously, three different grades were contemplated for CAPA: proven, probable, and possible [5].

(A). Proven CAPA is defined as pulmonary or tracheobronchial infection. It is proven by either or both histopathological and direct microscopic detection of fungal elements morphologically consistent with *Aspergillus* spp. that show invasive growth into tissues with associated tissue damage, either alone or in combination with *Aspergillus* recovered by culture, detected by microscopy in histology studies, or detected by PCR from material obtained by sterile aspiration or biopsy from a pulmonary site.
(B). Probable CAPA involves the presence of tracheobronchial ulceration, nodule, pseudo-membrane, plaque, or eschar, alone or in combination, on bronchoscopic analysis and mycological evidence by positive culture for *Aspergillus*. Probable pulmonary CAPA also required a pulmonary infiltrate or nodules, preferably documented by chest CT, or cavitating infiltrate (not attributed to another cause), or both, combined with mycological evidence, serum GM index > 0.5, and clinical criteria. Detection of GM in NBL is considered to be evidence for CAPA.
(C). Possible CAPA: In the setting of COVID-19 and in view of the challenges related to CAPA diagnosis, this category requires pulmonary infiltrate or nodules, preferably documented by chest CT, or cavitating infiltrate (which is not attributed to another cause) in combination with mycological evidence (e.g., microscopy, culture, or GM, alone or in combination) obtained via NBL, including those who have undergone NBL to obtain mycological evidence.

### 2.3. Definition of Diabetes, Obesity, and Hypertension

Diabetes was defined, diagnosed, and classified according to the ADA criteria [25]. Diabetes care in hospitalized patients was administered according the Standards of Medical Care in Diabetes 2022 guidelines [26]. Patients were considered to have obesity when they had a body mass index (BMI) of ≥30 kg/m^2^, and they were classified as having obesity class I, II, or III when their BMI was 30–34.9 kg/m^2^, 35–39.9 kg/m^2^, or ≥40 kg/m^2^, respectively [27].

In accordance with the ISH guidelines, hypertension was defined as systolic blood pressure (SBP) ≥140 mmHg and/or diastolic blood pressure (DBP) ≥90 mmHg, following repeated examination [24]. Hypertension classification was considered as high-normal, grade 1, and grade 2 according to the presence of other risk factors, hypertension-mediated organ damage, and previous disease [24].

## 3. Case Report

### 3.1. Case Report 1

**Figure 2:**
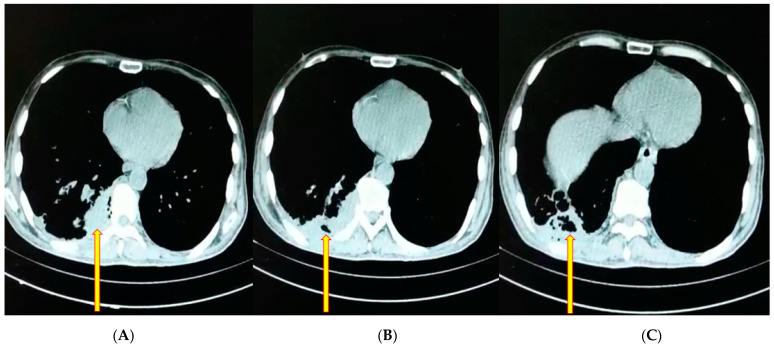
Computed tomography of the chest. (**A**) Axial computed tomography (CT) in a 51-year-old man showing right lower lobe nodules and opacities, bilaterally. (**B**,**C**) CT images showing necrotizing pneumonia with air picture within the pulmonary infiltrate, indicated by arrows. These features are relevant to a necrotic lung fragment in the angio-invasive form of pulmonary aspergillosis and co-morbid COVID-19 (CAPA), pulmonary tuberculosis, and type 2 diabetes mellitus with moderate diabetic ketoacidosis.

A 51-year-old male farmer was admitted to hospital with T2DM of nine years evolution. He had been treated previously with metformin 850 mg three times daily and glibenclamide (5 mg) three times daily. He expressed no contact with patients with COVID-19, and he was not vaccinated against SARS-CoV-2/COVID-19. Six months before hospital admission, he had lost weight (15 kg). Starting one week earlier, he had been experiencing COVID-19 symptoms: fever 38.3 °C, anosmia, dysgeusia, throat and chest pain, cough and sputum, polypnea, and general fatigue. A nasal swab for SARS-CoV-2 screening was conducted, and viral detection was carried out using RT-PCR with a positive result. At hospital admission, he was 66 kg in weight and 1.67 m in height (BMI was of 23.6 kg/m^2^). Three days before admission to the hospital, his general condition and oxygenation worsened, and on the second day bilateral ground-glass attenuations and necrotizing pneumonia, in his right lower lobe, appeared on his chest CT (Figure 2A–C).

Blood gas analysis revealed pH = 7.08, PaO_2_ = 74, PaCO_2_ = 17, HCO_3_ = 5.3, and lactate = 84 mm, the criteria for moderated DKA. Laboratory findings on the day of admission revealed a low peripheral white blood cell count, 3.7 × 10^9^/L, but non-neutropenic (61%), a total neutrophil count of 2.257 × 10^9^/L, platelets (310 × 10^3^/mL), and hyperglycemia (554 mg/dL). Dexamethasone (8 mg/day) was administered for 10 days, along with enoxaparin (60 mg/daily/30 days subcutaneous) therapy. Metabolic control was started with basal glargine insulin 30 UI once daily, plus bolus correction regimen with subcutaneous rapid-acting insulin before meals for a target glucose range of 140–180 mg/dL (7.8–10 mmol/L) according to guidelines for diabetes care in the hospital [27].

A BAL Acid-Fast Bacilli (AFB) smear and Gene Xpert MTB/RIF assay were positive without rifampicin resistance. His Mantoux test was 10 mm positive. Serum GM by radioimmunoassay (RIA) was 2.2 ng/mL (reference cut off level: DO >1.5). Based on the 2020 ISHAM/ECMM consensus definitions by clinical examination and the findings of laboratory and imaging investigations, a diagnosis of mild COVID-19 and possible CAPA with pulmonary Mtb co-infection in a Mexican male with T2DM was established.

The patient was treated for TB with first-line anti-tubercular drugs by DOTS (Direct Observed Therapy) as proposed by the WHO [28], including isoniazid, rifampicin, pyrazinamide, and ethambutol, and likewise with voriconazole (200 mg twice daily) for concomitant pulmonary CAPA. Metabolic control of T2DM was conducted with intermediate-acting insulin. The patient improved clinically, became afebrile, and had a favorable outcome. He is currently in the support phase of therapy for pulmonary TB, for CAPA and with metabolic control.

### 3.2. Case Report 2 

**Figure 3:**
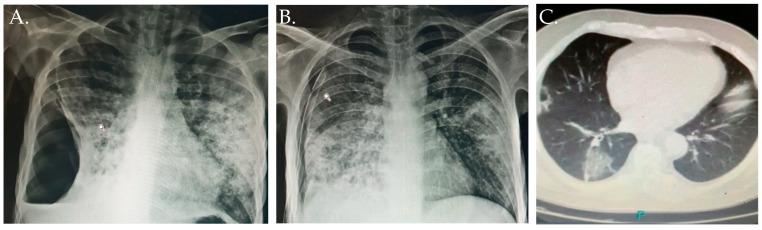
Chest radiography studies. (**A**,**B**) shows chest radiography study of Case 2. (**A**) Chest radiography of a 31-year-old male showed many small, thick-walled cavities bilaterally; evidence of perilesional ground glass haziness with air space opacities, right pneumothorax about 40%, pleural effusion, and many fibro-parenchymal opacities; a right chest drain tube was inserted (**B**). (**C**) shows a chest CT scan in a 67-year-old male (Case 3) revealing a right cavitary lesion, evidence of perilesional ground glass bilaterally with the largest lesion in the right lower lobe, and consolidative pulmonary opacities.

A 31-year-old male was admitted to hospital with complaints of cough lasting four months (sputum not specified), bed rest and weight loss unknown, loss of appetite for two weeks, shortness of breath for two weeks, and low-grade fever for one week. He had a diagnosis for T2DM eight years earlier and was irregularly taking oral antihyperglycemic agents and intermediate-acting insulin. His vaccination status against COVID-19 was unknown. In May, he tested positive for SARS-CoV-2/COVID-19 by RT-PCR. There was no history of TB or any COVID-19 cases among his contacts, and there was no history of smoking or any other substance abuse. His BMI was 19.4 kg/m^2^. There was no cyanosis, lymphadenopathy, or edema, he had tachypnea and breath sounds diminished in the right hemithorax. To establish a diagnosis, initially he was advised a chest axial CT and sputum culture for AFB. His chest CT (Figure 3A,B) showed many small, thick-walled cavities bilaterally, evidence of perilesional ground glass haziness with air space opacities, right pneumothorax about 40%, and pleural effusion; many fibro-parenchymal opacities and several nodules were also seen in both lungs; a right chest drain tube was inserted (Figure 3B).

Ziehl-Neelsen staining of the sputum was positive for acid-fast bacilli as 3+. Further, a Gene Xpert^®^ MTB/RIF assay detected Mtb with no resistance to rifampicin. His Montoux test was 15 mm. Pleural fluid was found to be exudative with an adenosine deaminase level of 43.5 U/L (cut off level <30 U/L). Many fibro-parenchymal opacities were also seen in both lungs. His fasting blood glucose levels were 151 mg/dL, and a test for serum GM was in the upper standard of diagnosis, with a titer of 2.89 ng/mL (cut off level DO >1.5). He suffered right leg deep venous thrombosis (DVT) diagnosed by Doppler US, a D-dimer level of 815 ng/mL, and a ferritin concentration of 1585 ng/mL.

Based on the 2020 ISHAM/ECMM consensus definitions patient examinations and the findings of laboratory investigations and imaging studies, a diagnosis of mild COVID-19 and possible CAPA with pulmonary TB co-infection in a Mexican male with T2DM was established. He was started on four-drug anti-tubercular therapy (ATT) by DOTS as per his weight with isoniazid, rifampicin, pyrazinamide, and ethambutol. Because of the diagnosis of concomitant pulmonary CAPA, we administrated voriconazole (200 mg twice daily). Metabolic control treatment was conducted using glargine insulin (20 U/daily subcutaneous). Treatment for DVT was with enoxaparin (40 mg/twice/daily). However, after 8 days, he requested his voluntary discharge from the hospital. He died 28 days after admission. According with the Mexican General Law of Health and the Guidelines for general and mass management of corpses due to COVID-19 (SARS-CoV-2), the clinical or pathological necropsy of this patient was not requested because the hospital facilities in where the patient was hospitalized did not have the conditions for the necropsy to be performed in a safe environment [29].

### 3.3. Case Report 3

A 67-year-old male grocery store worker was admitted to hospital; he had been diagnosed with T2DM and hypertension nine years earlier and was taking oral hypoglycemic drugs, anti-hypertensive drugs, and intermediate-acting insulin, but his control was irregular. He had been undergoing treatment for pulmonary and genital TB disease for three weeks. He was diagnosed for TB by histopathology studies and treated with ATT by DOTS as proposed by the WHO [28], including isoniazid, rifampicin, pyrazinamide, and ethambutol for 9 months. His BMI was 17.6 kg/m^2^ (low weight).

Fifteen days earlier, the patient had begun to suffer a low-grade fever and regular coughing episodes; these episodes were associated with thick, blood-tinged sputum, breathlessness aggravated by walking and sometimes at rest, and loss of appetite. At the time of admission, he was not vaccinated against SARS-CoV-2. On the eighth day of his hospital admission, his RT-PCR for SARS-CoV-2/COVID 19 was positive. On examination, he had reduced breath sounds over the left lower lung zone with coarse crackles. His vital signs were within the normal range, and oxygen saturation in air was 78%. The rest of the systemic examination revealed nothing of note. His fasting blood glucose levels were 230 mg/dL, serum urea was 44.2 mg/dL, and serum creatinine was 1.2 mg/dL.

His chest CT scan revealed right cavitary lesions and evidence of perilesional ground glass bilaterally, with the largest lesion in the right lower lobe, and consolidative pulmonary opacities (Figure 3C). Serum GM (RIA) was positive with a titer of 1.8 ng/mL (cutoff level DO >1.5). On the basis of the 2020 ISHAM/ECMM consensus definitions, the clinical examination and the findings of laboratory investigations and imaging studies, a diagnosis of mild COVID-19 with pulmonary and genital TB and possible CAPA co-infection in a Mexican male with T2DM and hypertension was established. During hospitalization his treatment was intermediated insulin 15 UI once daily, plus bolus correction regimen with subcutaneous rapid-acting insulin before meals for a target glucose range of 140–180 mg/dL (7.8–10 mmol/L) and administrated voriconazole (200 mg twice daily) [27]. The patient’s symptoms and oxygen requirements gradually improved, and he was discharged home after 15 days because of improvement. He continues with DOTS treatment, anti-hypertensive drugs, voriconazole and metabolic control of T2DM.

## 4. Discussion

In this study, we aimed to provide descriptive information about three patients with ongoing COVID-19 and co-infection with pulmonary TB, and possible CAPA, each one with comorbidities such as decompensated T2DM with DKA and SAH.

In all the patients in our study, NCDs (diabetes mellitus and hypertension) and three infectious diseases (COVID-19, TB, and possible CAPA) converged. Because these patients had no regular treatment for each NCD, we hypothesize that there were common intrinsic factors that predisposed them to infection by intracellular pathogens and/or opportunistic fungal infections. This internal condition is the initial failure to produce IFN-γ, a phenomenon possibly related to genetic/immunologic failure along the IFN-γ/IL-12/IL-23 axis in a pattern called “Mendelian susceptibility to Mycobacterial diseases,” a type of primary immunodeficiency, of which opportunistic infections such as Mtb, CAPA, and other intracellular pathogens can take advantage [30,31]. Moreover, immunosuppressive therapy, such as the corticosteroids used alone in our patients, has been used in subjects with mild/severe COVID-19 to diminish and control hyperinflammation and CSS [6,32]; however, their principal diseases (i.e., uncontrolled T2DM/hypertension) worsened these infectious health conditions, favoring the proliferation of infectious pathogens and causing major damage. SARS-CoV-2 induces cellular death of pneumocytes by pyroptosis and apoptosis in endothelial cells [33], thickening of the alveolar wall, and worsening of hypoxia. In addition, uncontrolled intracellular proliferation of Mtb by failure of the IFN-γ IL-12/IL-23 axis and TNFα reduces acidification of lysosomes with less destruction of Mtb. Mtb accumulation at intracellular sites may be attributed to decreased or absent activity of vacuolar proton ATPase [34] and it is probable that the abuse by prescription and auto-medication of people with proton-pump inhibitors (PPI) worsened the problem. The death of different immune cells (e.g., macrophages, neutrophils, and T cells) may release hydrolytic enzymes that, according to our hypothesis, destroy pulmonary tissues, forming cavities, bronchiectasis, and pneumonitis, because the bacilli itself have neither toxins nor hydrolytic enzymes. Cavitary and pneumothorax lung lesions and pleural effusions are usually related to Mtb disease and are uncommon in COVID-19 pneumonia. *Aspergillus* is a fungus with a wide spectrum of pulmonary diseases, ranging from a noninvasive to an invasive infection, and, because of its angio-trophism, may form microthrombus and pulmonary embolism with an imbalance in the ventilation/perfusion ratio, which worsens hypoxia. On the other hand, *Aspergillus* may cause pulmonary infarction and cavitary lesions. These are associated with a high fungal and bacillary Mtb load, which may complicate pulmonary TB and make the patient highly contagious.

After an exhaustive search, there are no published reports of studies merging COVID-19, CAPA and TB; however, in Table 1 we present a summary of basic data from some of the most relevant variables taken from recent studies of COVID-19 and CAPA [35,36,37] or COVID-19 and TB [38,39,40], respectively.

According to the results of our literature research, the three cases in our study are rare and are the first of their type reported from Mexico and in the world in the current syndemic of COVID-19. Two of the patients survived and are in therapy with anti-Mtb DOTS, voriconazole treatment for aspergillosis, and treatments for their concomitant diseases with drugs provided by the Mexican public health system. They are also under control, with liver enzymatic testing and electrocardiography because of the possible deleterious side effects of anti-Mtb and voriconazole treatment, such as drug hepatitis damage or long QT syndrome [41]. Regarding our third patient, who did not survive, a male 31-year-old, he was the youngest of the studied patients, but it is unclear why he died. We theorized that he did not continue with treatment and/or that his immune system was strongest, and he could have had a hyperinflammatory syndrome caused by CSS, with the highest levels of inflammatory molecules, higher than those of the other patients, who were older and thus could have had a more senescent immune system.

Although TB is one of the main causes of mortality worldwide, its diagnosis in many low-resource, high-burden countries still relies on microscopic examination of sputum smears, which lacks sensitivity and specificity. The gold standard for TB diagnosis by culture of Mtb takes several weeks to become positive, with additional tests required for final identification. The most commonly used proportion method on Lowenstein–Jensen medium or Middlebrook agar requires 3–6 weeks to yield results. Here, molecular diagnostic tests such as Gen Xpert and Gen Xpert Ultra take a principal place in the diagnosis of treatment-sensitive and treatment-resistant TB.

Limited diagnostic procedures, such as bronchoscopy examinations (bronchoscopy with BAL remains the cornerstone of CAPA diagnosis) and measurements of GM antigen and BDG, are very expensive. These limitations contribute to diagnostic difficulties in developing countries such as Mexico, where it is highly probable that CAPA may be underdiagnosed. In our study, bronchoscopy was not performed; instead, for CAPA diagnosis, we used the 2020 ISHAM/ECMM consensus definitions. It is important to emphasize that definitions such as those published by the EORTC/MSGERC are recommended only for research purposes and should not be used for clinical decision making. We believe that a more pragmatic approach to the diagnosis of CAPA in the setting of a patient with severe COVID-19 pneumonia in critical care would be to combine ≥2 of the following mycological criteria: 1. GM detection from serum/BALF/ETA, because the hallmark pathologic feature of IPA is angioinvasion and GM is a marker of hematogenous dissemination of the microorganism. GM-index threshold greater than 1.5 (as was seen in our patients) generate specificities greater than 90% and positive likelihood ratios (i.e., >10) that have been sufficient to confirm CAPA in other studies [5]. For mycological tests and cutoffs, we aimed to comply with other IPA case definitions, if possible (i.e., Verwejj et al., who state that IAPA diagnosis requires pulmonary infiltrate, preferably documented by chest CT, and at least one of the following criteria: serum GM >0.5 and GM in BAL >1) [5,42,43]. However, a specific marker for tissue invasion that can distinguish *Aspergillus* respiratory tract colonization is urgently needed [13]. 2. Isolation of *Aspergillus* spp. from BALF/ETA/sputum. 3. Serum BDG detection, (a pan-fungal biomarker). 4. Detection of *Aspergillus* DNA by RT-PCR from blood or respiratory samples [4,5]. The typical “halo sign” associated with IPA in patients with neutropenia is uncommonly seen in non-neutropenic patients with IPA, where radiological imaging may show varying patterns from multiple pulmonary nodules to various non-specific findings, which include consolidation, cavitation, pleural effusions, ground glass opacities, tree-in-bud opacities, and atelectasis, as in our patients. High-resolution CT is preferred to other imaging methods, such as chest radiographs. Because our patients had ongoing Probable CAPA by serology, imaging and clinical criteria (2020 ISHAM/ECMM consensus definitions), none of them received piperacillin–tazobactam for a false-positive in GM test [4,5]. Moreover, our patients were immunocompromised to different degrees according to their NCDs, and people with COVID-19 and concurrent diabetes have worse prognoses and mortality rates [6,22,44,45].

Finally, it is important to note that therapies for COVID-19 in patients with diagnosed diabetes should involve adequate glycemic control [46]. This requires consideration of all potential implications that therapies for COVID-19 might generate [17,46]. Conversely, antiviral drugs (lopinavir and ritonavir) worsen glycemic control because they could lead to hyperglycemia. Because these drugs can cause hepatic and muscle toxicity, their use in combination with statins or in patients with fatty liver disease should be considered carefully. Moreover, it has been suggested that SARS-CoV-2 tropism for β-cells could cause acute impairment of insulin secretion or destruction of β-cells, resulting in de novo development of diabetes [17]. In the same way, overall, immunosuppressive therapy is an important therapeutic option for patients with severe COVID-19; however, it is also a risk factor for opportunistic infection [6], including that caused by Mtb, *Aspergillus* spp., mucormycosis, *Candida* spp., *Cryptococcus neoformans*, *Pneumocystis jiroveci* (carinii), cytomegalovirus, herpes simplex virus, *Strongyloides stercoralis*, and *Toxoplasma gondii* [6,32]. According to the above, the generation of reliable algorithms is urgently needed for treatment of people with COVID-19 and comorbidities, standardization of diagnostic procedures, and even the use of newly available pharmaceutical agents that target a critical component of inflammasome activation, signaling pathways leading to cellular pyroptosis, and the downstream cytokines, as a promising target for the treatment of severe COVID-19-associated diseases, COVID-19, and future viral diseases.

Study limitations: It is important to note that the main limitation of the study is that the diagnosis of aspergillosis was based on clinical, radiological images together with serum GM, but the fungus was not isolated. Another study limitation was that, in Zacatecas, Mexico, where the study was carried out, the hospital facilities do not have the requirements to carry out clinical or pathological necropsy due to COVID-19, which could have allowed us to obtain valuable complementary data. In spite of accordance with the 2020 ISHAM/ECMM consensus definitions, the patients could be classified as possible CAPA; additional studies will be necessary to establish the molecular and pathological basis of the convergence of SARS-CoV-2, *Aspergillus*, and Mtb in a syndemic context.

## 5. Conclusions

The three patients in this study had diagnoses of NCDs (T2DM and hypertension), each one with irregular treatment for each condition, together with three infectious diseases (COVID-19, pulmonary TB, and possible CAPA). These three cases are rare and are the first of their type reported from Mexico and worldwide. Because of the difficulty and cost of diagnostic tests (GM antigen and BDG), it is highly probable that CAPA may be underdiagnosed in our country. Considering the high prevalence of NCD, mainly T2DM, hypertension, and obesity, these individuals represent a large, vulnerable segment of the COVID-19 population and may be targets of other opportunistic infectious pathogens. The therapy for COVID-19 in patients with comorbidities and other infectious diseases requires consideration of all potential implications that these therapies might generate. COVID-19 meets the criteria to be considered a syndemic and, because of the gravidity of the actual situation, the generation of reliable algorithms, early diagnostics, standardization of classification criteria, and the selection of specific and personalized treatments for COVID-19-associated opportunistic infections, including CAPA, are necessary to improve outcomes in these kinds of patients.

## Figures and Tables

**Table 1:** Summary of studies reporting COVID-19 and CAPA or COVID-19 and TB.

COVID-19 and CAPA						
Study, total cases, reference	Mean age (±SD or IQR) and percentage by gender	CAPA total Percentage and by gender (x/n) if available	Comorbidities and other pre-existing condition Percentage from total CAPA	Anti-COVID-19 treatment Percentage from total CAPA	ANF treatment	Outcome
Case-control, 65 COVID-19 cases, Tim Fischer et al., 2022 [35].	64.8 (±9.5) years, M: 74% (48/65), F: 26% (17/65)	CAPA: 20% (13/65), M: 54% (7/13), F: 46% (6/13)	ST: 62% (8/13) BWT: 62% (8/13) COPD: 15% (2/13) H 62% (8/13) CHD: 46% (6/13) CVA: 31% (4/13) DM: 31% (4/13) H: 61.5% (8/13)	S: 100% (13/13) IMD: 15% (2/13) Antiviral 8% (1/13)	No use of antifungal was reported in this study	tendency for consolidation in early COVID-19 disease Bacterial superinfection 77% (10/13)
Retrospective study, 221 COVID-19 cases, Jon Salmanton et al., 2021 [36].	68 (IQR 58–73) years, Not specified gender	CAPA: 84% (186/221), M: 73% (135/186), F: 27% (51/186)	*Aspergillus fumigatus*: 66% (122/186) *A. niger:* 7% (13/186) *A. flavus:* 5% (10/186) *A. terrenus:* 3% (6/186) CHD: 51% (94/186) RF: 40% (74/186) DM 34% (64/186)	C: 53% (98/186)	ANF: 74% (137/186) AB: 19% (36/186) E: 12.9% (24/186) T: 62.9% (117/186)	Overall mortality 52.2% (97/186) Cause of death CAPA 17.2% (32/186) COVID-19 (27.4% (51/186)
Multicenter retrospective study, 218 COVID-19 cases, Raeseok Lee et al., 2022 [37].	62 (49–72) years, M: 53% (116/218), F: 46.7% (102/218)	CAPA: 5% (10/128), M: 50% (5/10), F: 50% (5/10)	COPD 20% (2/10) TB 0% (0/10) DM 0% (0/10) CKD 20% (2/10) CAD 10% (1/10) CVA 0% (0/10)	C: 100% (10/10)	No use of antifungal was reported in this study	Overall in-hospital mortality 12% (26/218), higher rate in CAPA In hospital mortality 50% (5/10)
**COVID-19 and TB**						
Study/total cases/reference	Mean age (± SD or IQR) and percentage by gender	TB Total Percentage ”n” and by gender (x/n) if available	Underlying diseases or pre-existing condition Percentage from total TB	Anti-COVID-19 treatment Percentage from total TB	ATT	Outcome
Cohorts study, 49 TB/COVID-19 cases, Tadolini M et al., 2020 [38].	48 (32–69) years, M: 82% (40/49), F: 18% (9/49).	TB before COVID-19: 53% (26/49), COVID-19 before TB: 29% (14/49); diagnosed within the same week: 18% (9/49); on the same day: 8% (4/49)	COPD: 17% (8/47) DM: 16% (8/49) HIV: 13% (6/48) RF: 10% (5/49) LD: 14% (7/49) AA: 20% (10/49) SMK: 41% (20/49)	Overall: 57% (28/49), HCQ: 78% (22/28), A-HIV-PI: 43% (12/28), AZ: 25% (7/28)	Standard first-line regimen: 76% (37/49). Second-line drugs: 16% (8/49)	Cured: 12% (6/49), Completed: 2% (1/49), On treatment: 76% (37/49), Died: 10% (5/49)
Retrospective observational study, 1073 COVID-19 cases, Gupta et al., 2020 [39].	40.59 (19–67) years Active TB: 36 (27–59.5) years Treated TB: 44 (28–51) years	Active/treated TB: 2% (22/1073), Active TB: 59% (13/22), F: 85% (11/13) Treated TB: 41% (9/22) F: 100% (9/9)	H: 18% (4/22) HT: 5% (1/22) DM: 14% (3/22) SD: 9% (2/22)	Any especial COVID-19 treatment was mentioned in this study	Conventional ATT: 92% (12/13) Conventional MDR: 8% (1/13)	Died 27% (6/22), Discharged 73% (16/22) Deaths attributed to COVID-19 co-infection
Secondary analysis of health records, 844 COVID-19 cases, Yuliia Sereda et al., 2022 [40].	43.5 (15.6) years M: 64% (540/844) F: 36% (304/844)	MTB detected: 6% (47/844) M: 77% (36/47) F: 23% (11/47)	HTB: 15% (7/47) HIV: 4% (2/47) DM: 4% (2/47) Other: 49% (23/47)	Any especial COVID-19 treatment was mentioned in this study	History of ATT: 25% (12/47)	Overall IHM: 2% (19/844) COVID-19: 2% (18/797) COVID-19/TB: 2% (1/47)

CAPA: COVID-19 Associated Pulmonary Aspergillosis. H: Hypertension. CHD: Chronic heart disease. CAD: Coronary Artery Disease. CLD: Chronic lung disease, CKD: Chronic Kidney Disease. Renal Failure: RF. COPD: chronic obstructive pulmonary disease. CVA: Cerebrovascular Disease. DM: Diabetes Mellitus. ST: Septal Thickening. BWT: Bronchial Wall Thickening. S: Steroids. C: Corticosteroid. IMD: Immunomodulating Drugs. AB: Amphotericin B. E: Echinocandins. T: Triazol. ANF: Antifungal. LD: Liver Disease. AA: Alcohol Abuse. SMK: Smoking. HCQ: hydroxychloroquine. AZ: Azithromycin. A-HIV-PI: Anti HIV Protease Inhibitor. M: Male. F: Female. ATT: Anti-TB Treatment. HT: Hypothyroidism. SD: Seizure disorder. MDR: Multidrug-Resistant. IHM: In Hospital Mortality HTB: History of TB.

## Data Availability

The data presented in this study are available on request from the corresponding author.
